# Case Report: MOGAD - a steroid-responsive autoimmune meningoencephalitis mimicking infection

**DOI:** 10.3389/fimmu.2025.1516178

**Published:** 2025-02-24

**Authors:** Yihui Goh, Priscillia Pei Shi Lye, Paul Anantharajah Tambyah, Derek Tuck Loong Soon, Nares Smitasin, Jonathan Jia Yuan Ong, Clement Hsiang Rong Yong, Yee Cheun Chan, David Tang, Kay Wei Ping Ng, Raymond Chee Seong Seet, Amy May Lin Quek

**Affiliations:** ^1^ Division of Neurology, Department of Medicine, National University Hospital, Singapore, Singapore; ^2^ Division of Infectious Diseases, Department of Medicine, National University Hospital, Singapore, Singapore; ^3^ Department of Medicine, Yong Loo Lin School of Medicine, National University of Singapore, Singapore, Singapore; ^4^ Infectious Diseases Translational Research Program, Yong Loo Lin School of Medicine, National University of Singapore, Singapore, Singapore; ^5^ Department of Diagnostic Imaging, National University Hospital, Singapore, Singapore; ^6^ Department of Diagnostic Imaging, Yong Loo Lin School of Medicine, National University of Singapore, Singapore, Singapore

**Keywords:** MOGAD, MOG-IgG, aseptic meningoencephalitis, cerebral cortical encephalitis, autoimmune

## Abstract

This case series reports three patients initially managed for presumed infectious meningoencephalitis, who were ultimately diagnosed with myelin oligodendrocyte glycoprotein associated disease (MOGAD). Their clinical presentations were strikingly similar to those of acute infectious meningoencephalitis, which posed a challenge to the initial diagnostic process. Notably, despite the absence of typical radiological changes associated with MOGAD, such as cerebral cortical encephalitis, these patients exhibited focal neurological and electroencephalographic changes. This series highlights the importance of considering MOG antibody testing in cases of aseptic meningoencephalitis, where early and accurate diagnosis can influence treatment decisions and patient outcomes in this steroid-responsive condition.

## Introduction

Myelin oligodendrocyte glycoprotein associated disease (MOGAD), diagnosed by the presence of MOG immunoglobulin-G (MOG-IgG), often presents with optic neuritis, transverse myelitis, and acute demyelinating encephalomyelitis. Recently, MOGAD has been increasingly recognized to present as cerebral cortical encephalitis (CCE) phenotype (1), observed in up to 7% of MOGAD (2). CCE is radiologically defined by its hallmark cortical FLAIR hyperintense lesions and overlying leptomeningeal enhancement (3, 4).

Meningoencephalitis presents a significant treatment challenge, often due to its elusive underlying causes. Although immune-mediated causes such as MOGAD respond favorably to immunotherapy, they are not routinely considered in the differential diagnosis of aseptic meningoencephalitis (5, 6). Here, we describe three patients with fever, headache and focal neurological symptoms. Initially managed as infectious meningoencephalitis, they were later diagnosed with MOGAD following MOG-IgG testing, highlighting the importance of this condition in the differential diagnosis.

### Patient 1

**Figure 1:**
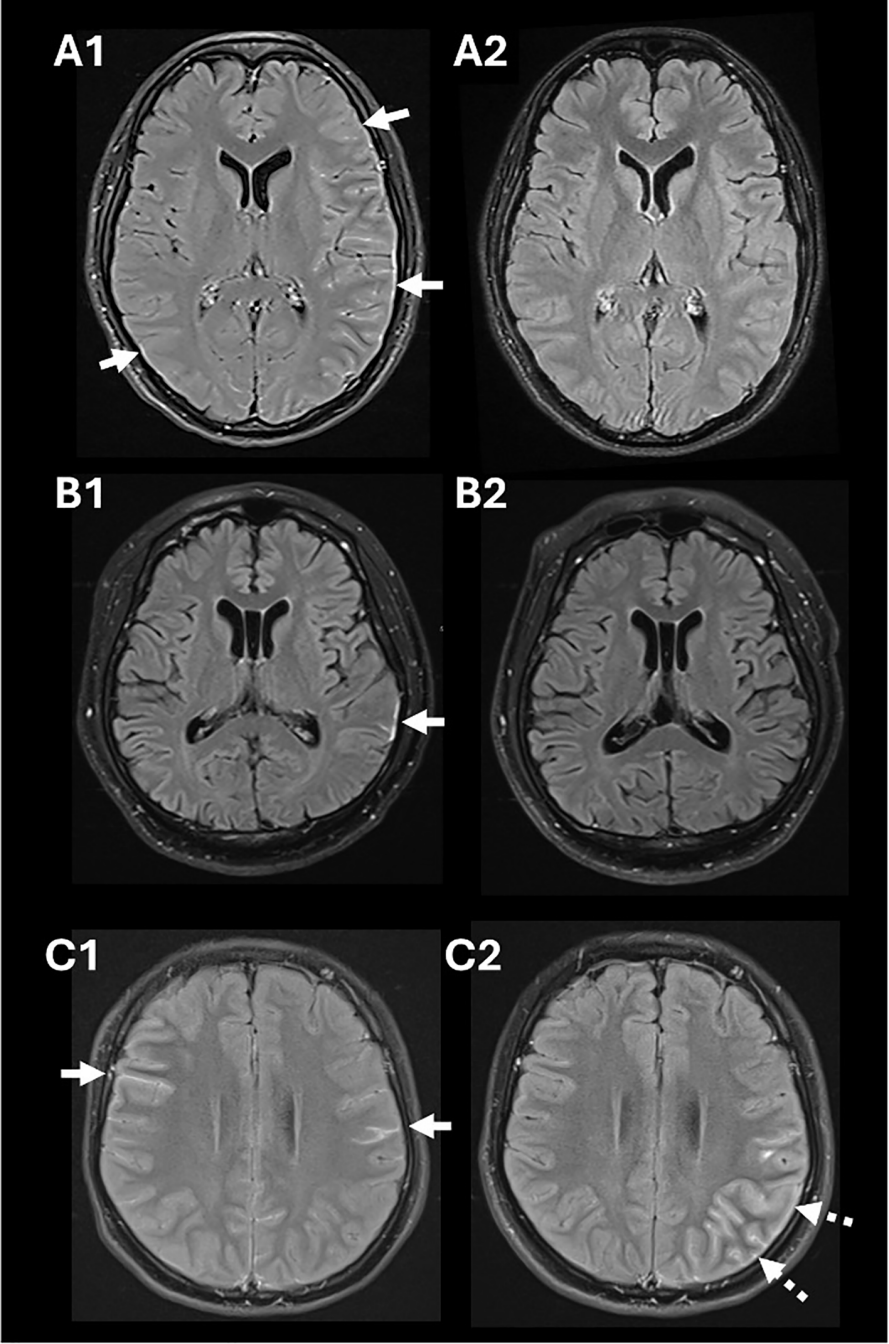
Salient findings in gadolinium-enhanced MRI of the brain using fluid attenuated inversion recovery (FLAIR) sequences. Diffuse leptomeningeal enhancement and cortical edema in Patient 1 (white arrows in **A1**) that resolved following immunotherapy **(A2)**. A focal area of enhancement was observed in the left parietotemporal region in Patient 2 (white arrow in **B1**) that resolved following immunotherapy **(B2)**. Leptomeningeal enhancement was detected in Patient 3 (white arrows in **C1**); imaging repeated when she relapsed revealed development of left cortical swelling and leptomeningeal enhancement (broken arrows in **C2**). There was no diffusion restriction associated with these MRI changes.

A 27-year-old woman, recently returned from rural India, presented with a 12-day history of subacute occipital throbbing radiating to the frontal region, high-grade fever (39.4°C) and generalized tonic-clonic seizures that prompted hospitalization. Examination revealed lethargy, nuchal rigidity, expressive dysphasia, right hemiplegia, and a non-blanching petechial rash. A non-contrast brain CT performed two weeks after headache onset was normal, but electroencephalography showed electrographic seizures originating from the left hemisphere. Her full blood count demonstrated mild leukocytosis (WBC 11.16 × 10^9^/L; normal 4.3–10.4) and mild lymphocytosis (3.74 × 10^9^/L; normal 1.21–3.56), with a normal platelet count (333 × 10^9^/L; normal 150–410). Cerebrospinal fluid (CSF) evaluation showed lymphocytic pleocytosis (WBC 367/µL; normal 0-5/µL, 85% lymphocytes), elevated protein levels (0.89g/L; 0.15-0.40) and CSF to serum glucose ratio of 0.47. Despite empirical treatment for infection with ceftriaxone, acyclovir, ampicillin and doxycycline, there was no clinical improvement. Subsequent magnetic resonance imaging (MRI) of the brain, performed on day 17 from headache onset, revealed left hemispheric leptomeningeal enhancement and cortical edema (**Figure 1A1**). A repeat CSF evaluation showed worsening pleocytosis (WBC 493/μL, 63% lymphocytes) with elevated CSF protein (0.6g/L, normal 0.15-0.40) and CSF to serum glucose ratio at 0.53. Given the MRI features, negative infectious evaluations (including Gram stain, culture, meningitis/encephalitis film array panels; **Supplementary Table 1**), and lack of response to antimicrobials, intravenous methylprednisolone (1g daily for 5 days) was initiated for a presumed autoimmune etiology. Her neurological symptoms significantly improved with immunotherapy. Intravenous immunoglobulin (IVIg) (0.4g/kg body weight for 5 days) was further administered for mild residual cognitive deficits. Subsequent live cell-based assays (CBA) detected MOG-IgG in both serum (FACS titer 1:100; normal <1:20) and CSF (IgG-binding index 21.4; normal, <2.5). She was discharged on oral prednisolone, tapered from 50 mg daily over 10 months, with azathioprine added to prevent relapse and support her rehabilitation. Minimal deficits in recall and processing speed improved, and she fully recovered clinically and radiologically over one year (**Figure 1A2**).

### Patient 2

A 51-year-old diabetic man presented with a subacute onset of fever (38.3°C), headache, speech difficulty, and drowsiness over six days. On examination, he exhibited dysphasia and an inability to follow one-step commands, with a Glasgow Coma Scale (GCS) of 12. He was initially treated for suspected infectious meningoencephalitis with intravenous ceftriaxone, ampicillin, and acyclovir. Electroencephalography revealed electrographic seizures originating from the left temporal region. CSF analysis showed a WBC count of 15/µL (lymphocyte predominance, 87%), elevated protein (1.56 g/l) and a CSF to glucose ratio of 0.61. Brain MRI revealed left temporo-parietal focal leptomeningeal enhancement (**Figure 1B1**). On day 9 of illness, he developed a mild cough. Testing confirmed SARS-CoV-2 infection, and he was treated with dexamethasone (6mg daily for 6 days) for presumed COVID-19-associated pneumonia. This resulted in improvement in his mentation, with slightly more coherent speech, and GCS of 15. Given his persistent neurological symptoms, intravenous methylprednisolone (1g daily for 3 days) was initiated for presumed COVID-19-associated encephalitis. This led to further neurological improvement. Subsequent testing confirmed the presence of MOG-IgG (FACS titer 1:100, normal <1:20). Following a tapering dose of prednisolone, he improved (**Figure 1B2**) and was able to return to work within two months.

### Patient 3

A 16-year-old woman presented with a subacute onset of fever (38.6°C), sharp right temporal headache, photophobia, and vomiting. The headache, which began gradually, peaked in intensity and was accompanied by confusion and left faciobrachial weakness. Brain MRI showed diffuse leptomeningeal enhancement (**Figure 1C1**). CSF analysis revealed lymphocytic pleocytosis (WBC 96/µL, 42% lymphocytes), slightly elevated protein (0.42 g/L) and a CSF to serum glucose ratio of 0.63. Despite antimicrobial treatment for presumed meningitis, her condition deteriorated, with increasing agitation requiring sedation. Infectious screens returned negative, and intravenous methylprednisolone was initiated, resulting in significant clinical improvement. MOG-IgG testing during her admission confirmed positivity via live CBA (FACS 1:100). She was discharged on a tapering dose of oral prednisolone. Two months following prednisolone discontinuation, she contracted COVID-19, which triggered a relapse of neurological symptoms, including headaches and right-sided motor automatisms. Repeat imaging revealed interval development of left cortical swelling and increased leptomeningeal enhancement (**Figure 1C2**). Her symptoms improved after resuming steroids, and she was started on mycophenolate mofetil, which she continues one year after her second presentation, with no further relapses.

## Discussion

The clinical presentations of these patients illustrate the diagnostic challenge of distinguishing MOGAD-associated meningoencephalitis from infectious causes. All three patients exhibited fever, headache and encephalopathy - symptoms commonly associated with intracranial infections. In Patient 1, recent travel to a Japanese encephalitis-endemic area in Asia contributed to a strong presumption of infection, later ruled out when her serology returned negative. She also developed a petechial rash, which ultimately proved unrelated. In Patient 2, initial concern for COVID-19 encephalitis delayed the consideration of MOGAD until suboptimal improvement and subsequent findings prompted a diagnostic shift. Notably, none of the patients displayed typical inflammatory features such as optic neuritis or transverse myelitis, further obscuring the diagnosis.

Certain features beyond a lack of response to antimicrobial therapy may help distinguish MOGAD encephalitis from infectious etiologies. Cortical FLAIR hyperintense lesions with overlying leptomeningeal enhancement represent a recognizable radiological hallmark of MOGAD-associated cerebral cortical encephalitis (CCE). However, these findings can be absent in the early stages (7, 8). In such cases, neurological signs, including dysphasia, focal motor weakness, or electroencephalographic changes, may precede radiological changes, providing important clinical clues to an underlying cortical process and warranting early consideration of MOGAD testing.

Meningoencephalitis has a broad differential diagnosis, and initial management typically targets potential infectious etiologies (9). However, in aseptic cases, when initial microbiological tests are negative, and clinical or radiological features suggest focal cortical involvement, early MOG-IgG testing should be considered. A definitive diagnosis of MOGAD requires a strongly positive MOG-IgG result, ideally via the live CBA method, given its superior specificity over the fixed assay (1, 10). In these three cases, all had definitively positive results using the live CBA. Nevertheless, a MOG-IgG titer of 1:100, carries an 82% positive predictive value (11) which, while robust, is not absolute. Thus, the clinical context, including neurological findings, imaging and treatment response, must guide the interpretation of MOG-IgG levels. For example, Patient 2 demonstrated cortical neurological changes, yet showed only focal leptomeningeal enhancement, a pattern less typical of MOGAD-CCE. Nonetheless, his strong MOG-IgG positivity, alongside improvement with immunotherapy, supported the diagnosis. In similar scenarios, high titer MOG-IgG can help diagnose MOGAD-CCE even in atypical presentations, but clinicians should remain vigilant and integrate all available clinical data before making a final diagnosis.

The prompt initiation of immunotherapy, comprising intravenous steroids followed by an oral tapering regimen (2), is essential for achieving optimal recovery in in MOGAD. Despite concerns that immunotherapy might trigger flare-ups in undiagnosed infectious causes of meningoencephalitis, there is no evidence to support this. In fact, corticosteroids are increasingly used as adjunctive treatment for certain bacterial and mycobacterial meningitis (12). Our patients’ rapid response to immunotherapy and subsequent negative microbiological results validated the rationality of this approach. To conclude, MOG-IgG testing should be included in the diagnostic workup of aseptic meningoencephalitis to facilitate timely diagnosis and treatment for this potentially reversible condition. Even when CCE is absent, clinical findings of focal cortical dysfunction, infective work ups and strong responses to steroids can guide physicians toward an autoimmune etiology.

## Data Availability

The original contributions presented in the study are included in the article/**Supplementary Material**. Further inquiries can be directed to the corresponding author.
