# Supplementary material for: Case Report: MOGAD - a steroid-responsive autoimmune meningoencephalitis mimicking infection
Source: Front Immunol. 2025 Feb 24;16:1516178. doi: 10.3389/fimmu.2025.1516178 (PMC11891045; doi:10.3389/fimmu.2025.1516178)
Supplement: Supplementary file 1 [file DataSheet1.docx]

**Supplementary Table: Investigation Results**

**Investigation Results of Patient 1**

| **Microbiological investigations**   \| Investigation \| Results \| Normal Range \| \| --- \| --- \| --- \| \| Dengue NS1 antigen \| Negative \| - \| \| Dengue IgM & IgG \| Negative \| - \| \| Dengue virus PCR \| Negative \| - \| \| Malarial parasite microscopy (3 sets) \| Negative \| - \| \| Blood cultures (3 sets) \| Negative \| - \| \| HIV Ab-Ag \| Negative \| - \| \| Syphilis screen \| Negative \| - \| \| Rickettsia/ orientia serology \| Negative \| - \| \| Chikungunya IgM & IgG \| Negative \| - \| \| Chikungunya virus PCR \| Negative \| - \| \| Japanese encephalitis virus IgM & IgG \| Negative \| - \| \| Japanese encephalitis virus PCR \| Negative \| - \| \| Toxoplasma gondii PCR \| Negative \| - \| \| Enterovirus PCR \| Negative \| - \| \| West Nile virus IgM & IgG \| Negative \| - \| \| Measles PCR \| Negative \| - \| \| Measles IgG \| Non-reactive \| - \| \| Brucella IgM \| **Reactive*** \| - \| \| Brucella IgG \| Negative \| - \| \| Mumps IgG \| Non-reactive \| - \| \| Rubella IgG \| **Reactive^#^** \| - \| \| Tetanus toxoid IgG \| **Positive^#^** \| - \| \| TB quantiferon \| Indeterminate \| - \| \| AFB smear and culture (2 sets, nasogastric aspirate) \| Negative \| - \| \| TB PCR (nasogastric aspirate) \| Negative \| - \| \| Zika virus PCR (urine) \| Negative \| - \| \| Respiratory virus multiplex (nasopharyngeal swab) \| Negative \| - \| |
| --- | --- | --- | --- | --- | --- | --- | --- | --- | --- | --- | --- | --- | --- | --- | --- | --- | --- | --- | --- | --- | --- | --- | --- | --- | --- | --- | --- | --- | --- | --- | --- | --- | --- | --- | --- | --- | --- | --- | --- | --- | --- | --- | --- | --- | --- | --- | --- | --- | --- | --- | --- | --- | --- | --- | --- | --- | --- | --- | --- | --- | --- | --- | --- | --- | --- | --- | --- | --- | --- | --- | --- | --- | --- | --- | --- | --- | --- | --- | --- | --- | --- | --- | --- | --- |

*****repeated 6 weeks later – Brucella IgM remained positive, IgG negative. Deemed to be false positive.

^#^Previously immunized against rubella and tetanus

| **Cerebrospinal fluid investigations**   \| Investigation \| Results on Day 1 of admission \| Results on Day 3 of admission \| Normal Range \| \| --- \| --- \| --- \| --- \| \| Opening pressure \| 17.5 cm H2O \| 15.0 cm H2O \| <25.0 cmH2O \| \| WBC count \| 367 (85% lymphocytes) /μL \| 493 (63% lymphocytes) /μL \| 0-5/μL \| \| Glucose \| 3.4 mmol/L (serum 7.3 mmol/L) \| 2.8 mmol/L (serum 5.3 mmol/L) \| 2.2-3.9mmol/L \| \| CSF to serum glucose ratio \| 0.47 \| 0.53 \|  \| \| Protein \| 0.89 g/L \| 0.60 g/L \| 0.15-0.40 g/L \| \| Gram stain and culture \| Negative \| Negative \| - \| \| Filmarray meningitis/encephalitis panel \| Negative \| - \| - \| \| Fungal smear and culture \| Negative \| Negative \| - \| \| HSV PCR \| Negative \| - \| - \| \| VZV PCR \| - \| Negative \| - \| \| Cryptococcal antigen \| Negative \| - \| <1 \| \| AFB smear and culture \| - \| Negative \| - \| \| TB PCR \| Negative \| - \| - \| \| Japanese encephalitis virus PCR \| - \| Negative \| - \| \| Cytology \| Inflammatory yield \| - \| - \| \| Flow cytometry \| Reactive lymphocytes. \| - \| - \| \| Oligoclonal bands \| - \| Matched serum and CSF oligoclonal bands \| - \| \| CSF autoimmune encephalopathy panel \| - \| Negative \| - \| \| CSF NMDA receptor antibody \| - \| Negative \| - \| \| CSF MOG antibody \| - \| **Positive, 21.3** \| <2.5 \|   **Other work-up for underlying etiology**   \| Investigation \| Results \| Normal Range \| \| --- \| --- \| --- \| \| Free thyroxine \| 12.5 pmol/L \| 9.0-19.1 pmol/L \| \| Thyroid stimulating hormone \| 0.52 mIU/L \| 0.35-4.94 mIU/L \| \| Blood and urine toxicology \| Negative \| - \| \| Beta-2- microglobulin \| 967 ug/L \| 970-2640 ug/L \| \| Lactate dehydrogenase \| 172 U/L \| 120-250 U/L \| \| Ferritin \| 164 ug/L \| 5-204 ug/L \| \| Anti-nuclear antibody \| <1:80 \| <1:80 \| \| Anti-ENA panel \| Negative \| - \| \| Erythrocyte sedimentation rate \| **15 mm/hr** \| 3-9 mm/hr \| \| High sensitive C-reactive protein \| 2.0 mg/L \| <5.0 mg/L \| \| C3 complement \| 95 mg/dL \| 83-193 mg/dL \| \| C4 complement \| 26 mg/dL \| 15-57 mg/dL \| \| Anti-ds-DNA \| <10 IU/ml \| <100 IU/ml \| \| ANCA \| Negative \| - \| \| Rheumatoid factor \| <20 IU/ml \| <30 IU/ml \| \| Angiotensin converting enzyme \| 10 U/L \| 16-85 U/L \| \| IgG4 subclass \| 37.9 mg/dL \| 2.4-121.0 mg/dL \| \| MOG antibody (FACS) (serum) \| **Positive, 1:100** \| <1:20 \| \| Serum autoimmune encephalopathy panel \| Negative \| - \| \| Serum NMDA receptor antibody \| Negative \| - \| \| Serum aquaporin-4 antibody \| Negative \| - \| |
| --- | --- | --- | --- | --- | --- | --- | --- | --- | --- | --- | --- | --- | --- | --- | --- | --- | --- | --- | --- | --- | --- | --- | --- | --- | --- | --- | --- | --- | --- | --- | --- | --- | --- | --- | --- | --- | --- | --- | --- | --- | --- | --- | --- | --- | --- | --- | --- | --- | --- | --- | --- | --- | --- | --- | --- | --- | --- | --- | --- | --- | --- | --- | --- | --- | --- | --- | --- | --- | --- | --- | --- | --- | --- | --- | --- | --- | --- | --- | --- | --- | --- | --- | --- | --- | --- | --- | --- | --- | --- | --- | --- | --- | --- | --- | --- | --- | --- | --- | --- | --- | --- | --- | --- | --- | --- | --- | --- | --- | --- | --- | --- | --- | --- | --- | --- | --- | --- | --- | --- | --- | --- | --- | --- | --- | --- | --- | --- | --- | --- | --- | --- | --- | --- | --- | --- | --- | --- | --- | --- | --- | --- | --- | --- | --- | --- | --- | --- | --- | --- | --- |

| **Investigation Results of Patient 2**   \| **Microbiological investigations**   \| Investigation \| Results \| Normal Range \| \| --- \| --- \| --- \| \| Blood cultures (1 set) \| Negative \| - \| \| HIV Ab-Ag \| Negative \| - \| \| COVID-19 RNA PCR \| **Detected** \| - \| \| \| --- \| --- \| --- \| --- \| --- \| --- \| --- \| --- \| --- \| --- \| --- \| --- \| --- \|   **Cerebrospinal fluid investigations**   \| Investigation \| Results on Day 2 of admission \| Results on Day 11 of admission \| Normal Range \| \| --- \| --- \| --- \| --- \| \| Opening pressure \| 14.0 cm H2O \| 13.0 cm H2O \| <25.0 cmH2O \| \| WBC count \| 15 (87% lymphocytes) /μL \| 15 (67% lymphocytes) /μL \| 0-5/μL \| \| Glucose \| 7.6 mmol/L (serum 12.4 mmol/L) \| 10.0 mmol/L (serum 19.3 mmol/L) \| 2.2-3.9mmol/L \| \| CSF to serum glucose ratio \| 0.61 \| 0.52 \|  \| \| Protein \| 1.56 g/L \| 1.40 g/L \| 0.15-0.40 g/L \| \| Gram stain and culture \| Negative \| Negative \| - \| \| Filmarray meningitis/encephalitis panel \| Negative \| - \| - \| \| Fungal smear and culture \| - \| Negative \| - \| \| HSV PCR \| Negative \| - \| - \| \| VZV PCR \| Negative \| - \| - \| \| Cryptococcal antigen \| Negative \| - \| <1 \| \| AFB smear and culture \| - \| Negative \| - \| \| TB PCR \| - \| Negative \| - \| \| Cytology \| - \| Lymphocytosis \| - \| \| Flow cytometry \| Reactive lymphocytes. \| Reactive lymphocytes. \| - \| \| Oligoclonal bands \| - \| Negative \| - \| \| CSF autoimmune encephalopathy panel \| - \| Negative \| - \|   **Other work-up for underlying etiology**   \| Investigation \| Result \| Normal Range \| \| --- \| --- \| --- \| \| Free thyroxine \| 11.3 pmol/L \| 9.0-19.1 pmol/L \| \| Thyroid stimulating hormone \| 1.23 mIU/L \| 0.35-4.94 mIU/L \| \| Lactate dehydrogenase \| 199 U/L \| 120-250 U/L \| \| High sensitive C-reactive protein \| 0.7 mg/L \| <5.0 mg/L \| \| MOG antibody (FACS) (serum) \| **Positive, 1:100** \| <1:20 \| \| Serum autoimmune encephalopathy panel \| Negative \| - \| |
| --- | --- | --- | --- | --- | --- | --- | --- | --- | --- | --- | --- | --- | --- | --- | --- | --- | --- | --- | --- | --- | --- | --- | --- | --- | --- | --- | --- | --- | --- | --- | --- | --- | --- | --- | --- | --- | --- | --- | --- | --- | --- | --- | --- | --- | --- | --- | --- | --- | --- | --- | --- | --- | --- | --- | --- | --- | --- | --- | --- | --- | --- | --- | --- | --- | --- | --- | --- | --- | --- | --- | --- | --- | --- | --- | --- | --- | --- | --- | --- | --- | --- | --- | --- | --- | --- | --- | --- | --- | --- | --- | --- | --- | --- | --- | --- | --- | --- | --- | --- | --- | --- | --- | --- | --- | --- | --- |

| **Investigation Results of Patient 3**   \| **Microbiological investigations**   \| Investigation \| Result \| Normal Range \| \| --- \| --- \| --- \| \| Dengue NS1 antigen \| Negative \| - \| \| Dengue IgM & IgG \| Negative \| - \| \| Blood cultures (2 sets) \| Negative \| - \| \| HIV Ab-Ag \| Negative \| - \| \| COVID-10 antigen \| Negative \|  \| \| \| --- \| --- \| --- \| --- \| --- \| --- \| --- \| --- \| --- \| --- \| --- \| --- \| --- \| --- \| --- \| --- \| --- \| --- \| --- \|   **Cerebrospinal fluid investigations**   \| Investigation \| Result on 1st admission \| Normal Range \| \| --- \| --- \| --- \| \| Opening pressure \| 19.0 cm H2O \| <25.0 cmH2O \| \| WBC count \| 96 (37% lymphocytes) /μL \| 0-5/μL \| \| Glucose \| 3.1 mmol/L (serum 4.9 mmol/L) \| 3.3-4.5mmol/L \| \| CSF to serum glucose \| 0.65 \|  \| \| Protein \| 0.48 g/L \| 0.15-0.40 g/L \| \| Gram stain and culture \| Negative \| - \| \| Filmarray meningitis/encephalitis panel \| Negative \| - \| \| Fungal smear and culture \| - \| - \| \| HSV PCR \| Negative \| - \| \| VZV PCR \| Negative \| - \| \| Cryptococcal antigen \| Negative \| <1 \| \| AFB smear and culture \| - \| - \| \| TB PCR \| Negative \| - \| \| Cytology \| Inflammatory yield \| - \| \| Flow cytometry \| - \| - \| \| Oligoclonal bands \| - \| - \| \| CSF autoimmune encephalopathy panel \| Negative \| - \| \| CSF NMDA receptor antibody \| Negative \| - \| \| CSF MOG antibody \| - \| <2.5 \|   **Other work-up for underlying etiology**   \| Investigation \| Result \| Normal Range \| \| --- \| --- \| --- \| \| Free thyroxine \| 14.0 pmol/L \| 10.0-14.3 pmol/L \| \| Thyroid stimulating hormone \| 0.93 mIU/L \| 0.35-4.94 mIU/L \| \| Anti-nuclear antibody \| 1:320 \| <1:80 \| \| Anti-ENA panel \| Negative \| - \| \| Procalcitonin \| <0.1 ug/L \| <0.5 ug/L \| \| High sensitive C-reactive protein \| 1.6 mg/L \| <5.0 mg/L \| \| C3 complement \| 134 mg/dL \| 83-193 mg/dL \| \| C4 complement \| 28 mg/dL \| 15-57 mg/dL \| \| Anti-ds-DNA \| <10 IU/ml \| <100 IU/ml \| \| ANCA \| Negative \| - \| \| MOG antibody (Serum via FACS) \| **Positive, 1:100** \| <1:20 \| \| MOG antibody (Cell binding assay) \| **Positive** \|  \| \| Serum autoimmune encephalopathy panel \| Negative \| - \| \| Serum NMDA receptor antibody \| Negative \| - \| \| Serum aquaporin-4 antibody \| Negative \| - \| |
| --- | --- | --- | --- | --- | --- | --- | --- | --- | --- | --- | --- | --- | --- | --- | --- | --- | --- | --- | --- | --- | --- | --- | --- | --- | --- | --- | --- | --- | --- | --- | --- | --- | --- | --- | --- | --- | --- | --- | --- | --- | --- | --- | --- | --- | --- | --- | --- | --- | --- | --- | --- | --- | --- | --- | --- | --- | --- | --- | --- | --- | --- | --- | --- | --- | --- | --- | --- | --- | --- | --- | --- | --- | --- | --- | --- | --- | --- | --- | --- | --- | --- | --- | --- | --- | --- | --- | --- | --- | --- | --- | --- | --- | --- | --- | --- | --- | --- | --- | --- | --- | --- | --- | --- | --- | --- | --- | --- | --- | --- | --- | --- | --- | --- | --- | --- | --- | --- | --- | --- | --- | --- | --- | --- | --- | --- | --- | --- |
